# The Single-Pitch Texel: A flexible and practical texture-rendering algorithm

**DOI:** 10.1093/pnasnexus/pgad452

**Published:** 2024-01-04

**Authors:** David A Burns, Roberta L Klatzky, Michael A Peshkin, J Edward Colgate

**Affiliations:** Department of Mechanical Engineering, Northwestern University, Evanston, IL 60208, USA; Department of Psychology, Carnegie Mellon, Pittsburgh, PA 15213, USA; Department of Mechanical Engineering, Northwestern University, Evanston, IL 60208, USA; Department of Mechanical Engineering, Northwestern University, Evanston, IL 60208, USA

**Keywords:** haptics, virtual textures, tactile codec

## Abstract

As the number of applications for tactile feedback technology rapidly increases, so too does the need for efficient, flexible, and extensible representations of virtual textures. The previously introduced Single-Pitch Texel rendering algorithm offers designers the ability to produce textures with perceptually wide-band spectral characteristics while requiring very few input parameters. This paper expands on the capabilities of the rendering algorithm. Diverse families of fine textures, with widely varied spectral characteristics, were shown to be rendered reliably using the Texel algorithm. Furthermore, by leveraging an assistive algorithm, subjects were shown to consistently navigate the Texel parameter space in a matching task. Finally, a psychophysical study was conducted to demonstrate the rendering algorithm’s resilience to spectral quantization, further reducing the data required to represent a virtual texture.

**Significance Statement**

The growing demand for high-quality multimedia transferred through global communication networks necessitates reliable and efficient techniques to compress such data with as little perceptual effect as possible. While well-established lossy digital formats exist for media in the domains of visual (e.g. JPEG) and audial (e.g. MP3) media, no such format exists for tactile (touch-sense) media. This work investigates one means of achieving a data-efficient, lossy virtual tactile texture representation, with the intention of enabling the transfer of touch-sense media through an interconnected network devices utilizing haptic feedback technology.

## Introduction

The addition of touch to the well-established modalities of vision and hearing as a viable avenue of virtual information transfer is rapidly becoming a reality in a wide variety of applications (1). Spurred by the advent of myriad technologies capable of producing artificial touch stimulation (2), it is reasonable to expect the prevalence of tactile feedback mechanisms to become increasingly prevalent with time.

One motivating factor for development of touch-enabled technology is the *Tactile Internet*, a hypothetical network structure with latency low enough to enable “real-time” interactions (3). Such an infrastructure would, among other benefits, allow for remote operation of machinery, equipment, and specialized tools by subject-matter experts, effectively expanding the reach of specialized skill sets worldwide. The advent of 5G communication networks, and development of 6G, both cast the Tactile Internet as a quickly approaching reality (4).

A critical obstacle toward achieving the Tactile Internet is devising haptic codecs capable of compressing touch-based data into a format that is simultaneously compact enough for rapid transmission while still containing all perceptually relevant information (5). Such codecs have received ample attention in recent research (6), with varied approaches leveraging strategies like a linear prediction scheme (7), discrete wavelet transforms (8), and quantization of signal spectral component amplitudes (9) to reduce the data load of a recorded tactile signal without affecting the user’s experience. Notably, haptic codecs like these function by starting with a maximum-data signal and apply compression by removing aspects of the signal that are not perceived by the user.

An alternate path toward achieving low-data-load tactile signals is building a virtual texture from elementary tactile features, adding complexity until perceptual similarity to the target reference is achieved. This approach is apparent in Hassan et al. (10), where the researchers deliberately generated new virtual textures by connecting psychophysical perceptual space and the space defined by measured tactile quantities (like normal force and swipe velocity). While this technique is effective at generating new realistic-feeling virtual textures, the need to use previously acquired data may limit the algorithm’s ability to render an arbitrary virtual texture conceived by a texture designer, especially those that cannot be represented by a physical, measurable texture.

## Background

### Surface haptics technology

Although generating virtual texture stimuli for perception by a user can be accomplished through a variety of means (graspable tool-based, wearable, moving pin arrays, etc.) (1), the ubiquity of touchscreen-based devices in the modern world suggests that focusing on the interaction between a bare fingertip and a flat device surface represents a viable path toward deploying touch-sense feedback on as many existing devices as possible (2). With this goal in mind, a logical focus is Surface Haptics: technology with the core intent of providing touch-sense stimuli to the user via control of the forces between the user’s fingertip and the device’s active surface (11). The control of tangential (in-plane) force on the fingertip has particular utility in reproducing the feedback useful for texture discrimination (12).

In this study, the tangential force between the flat device surface and the user’s fingertip is controlled via ultrasonic friction modulation, leveraging the effect of reduction in sliding friction along a surface by applying normal (out-of-plane) vibrations at ultrasonic frequencies (>20 kHz) with displacement on the order of a few micrometers (13). This reduction in friction has been established as dynamic levitation caused by both the intermittent contact between the asperities of the fingertip and the vibrating surface (14, 15) as well as the so-called squeeze film of air trapped beneath the fingertip (16, 17).

**Fig. 1.**
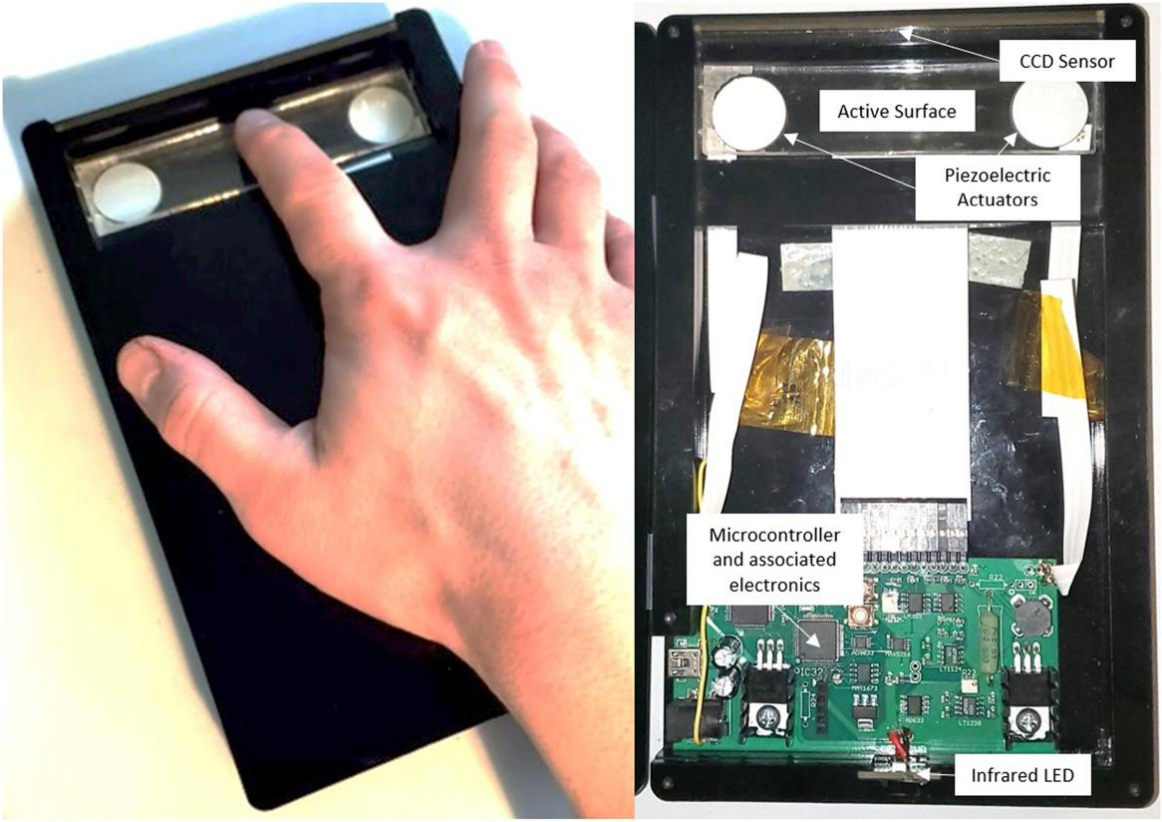
TPaD surface haptics device.

To utilize ultrasonic friction reduction for the purpose of virtual texture rendering, the primary author’s laboratory developed the Tactile Pattern Display (TPaD) (pictured in Fig. 1), combining optical finger position sensing with a borosilicate glass active surface vibrated out-of-plane at 34 kHz using piezoelectric actuators (18). One-dimensional virtual textures are stored on an on-board microcontroller (PIC32) in the form of a 19,200-element array. Each element gives the relative numerical amplitude of the carrier signal, which controls the magnitude of friction reduction. During playback, the amplitude of vibration is set by the value assigned to the element corresponding to the current finger position at a refresh rate of 8,333 Hz.

### Texel rendering

The Single-Pitch Texel-rendering algorithm, here referred to as *Texel rendering*, was introduced in Burns et al. (19) and demonstrated to efficiently recreate multifrequency virtual textures using a notably low number of input parameters. The rendering algorithm is summarized here.

**Fig. 2.**
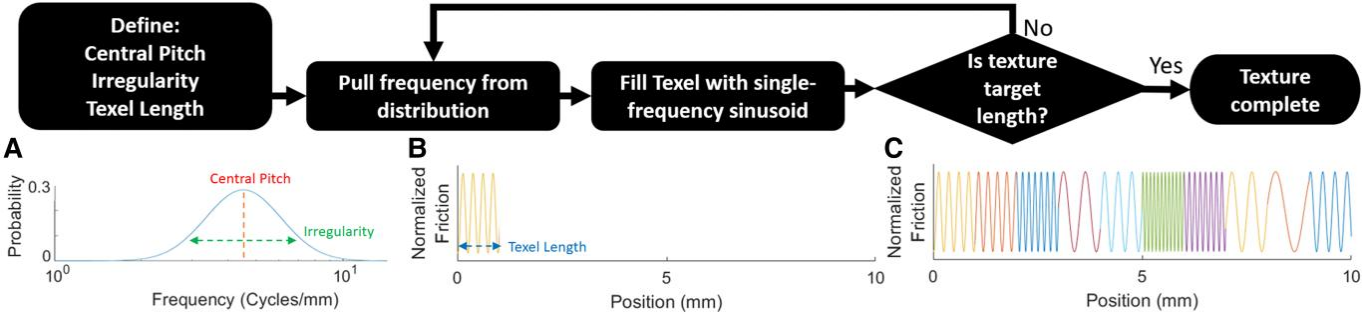
Flowchart depicting rendering algorithm. A) Texel frequency distribution, with “Central Pitch” (4.5 cycles/mm ≈6.5 dB) and “Irregularity” (0.1 cycles/mm =  −10 dB) parameters indicated. B) First texel with stochastically drawn frequency using a 1-mm texel length. Note that the starting phase of oscillation of the first texel is chosen randomly. C) Nine additional texels with stochastically drawn frequencies. Note that the phase of oscillation is continuous between the borders between adjacent texels. Adapted from a figure in Burns et al. (19) with permission from the authors and copyright holder (© 2021 IEEE).

Three parameters are first chosen by the texture designer: the physical length of a single Texel, the spectral mean of the pitch distribution (“Central Pitch”), and the spectral width (SD) of the pitch distribution (“Irregularity”). These parameters are used to build a pitch distribution that is Gaussian in log-frequency space. Starting at the left end of the texture, a value is drawn from the pitch distribution and used as the frequency for the Texel’s “pitch,” that is, a sinusoidal oscillation filling a single Texel length (initially with random starting phase of oscillation). This process is repeated for additional Texels, continuing to the right on the display (with phase of oscillation continuous across the borders of adjacent Texels) until the target texture length is achieved. This process is summarized in Fig. 2. For this study on fine-scaled textures, the amplitude of oscillation is held constant throughout. In future work, amplitude modulation will be used to introduce coarse texture features.

**Fig. 3.**
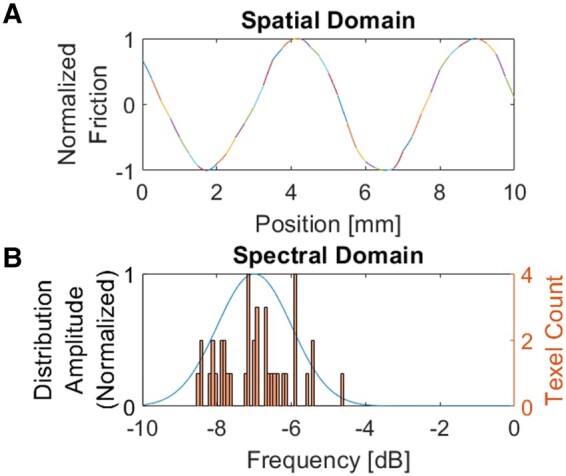
Single-Pitch Texel rendering of features with wavelength greater than the chosen Texel length. In this example, 40 Texels of length 0.25 mm render a texture with Center Frequency 0.2 cycles/mm (≈−7dB) and Irregularity 0.1 cycles/mm (=−10 dB). A) Spatial domain representation of texture. Normalized friction for each Texel displayed in alternating line styles for clarity. B) Spectral domain representation of texture. The underlying Texel frequency distribution (left axis) is overlayed with a histogram of individual Texel pitch values used in the rendering (right axis).

The requirement of continuous phase of oscillation at the borders between adjacent Texels allows for textural features to be rendered with wavelength greater than the chosen Texel length. As demonstrated in Fig. 3, a texture with features of oscillation frequency between 0.1 and 0.4 cycles/mm (that is, features with wavelength between 2.5 and 10 mm) can be reproduced with Texels of length 0.25 mm, as each Texel’s ending phase must match the starting phase of the next Texel. In this way, several adjacent Texels can form features with wavelengths well beyond the chosen Texel length.

Due to the log-scale nature of the input parameters of Central Pitch and Irregularity, in the remainder of this work, we will report both in units of dB with a reference value of 1 cycle/mm, abbreviated herein as simply “dB.” Values are computed using $X_{\mathrm{dB}}=10{\text{log}}_{10}(X_{\mathrm{cpm}}/(1\;\mathrm{cycle}/\mathrm{mm}))$, where $X_{\mathrm{dB}}$ and $X_{\mathrm{cpm}}$ are a parameter value in dB and cycles/mm, respectively. (Here, the power-quantity formula for decibels is used for the convenience of defining 10cpm=10 dB.)

In Burns et al. (19), the Texel-rendering algorithm was shown to efficiently recreate the sensation of a virtual texture with multiple frequency components present at all positions, provided that a texel length below 1 mm was used. Furthermore, the spectral parameters of distribution location and width were reliably tuned by participants to recreate reference textures, thereby representing effective dimensions for texture design.

In this work, we further test the utility of this rendering algorithm through matching tasks with a variety of reference textures encompassing a diverse set of wide-band spectral characteristics. Families of textures investigated included superpositions of multiple sinusoidal signals, spectrally shaped white noise signals (so-called *colors of noise*), and periodic nonsinusoidal waveforms. Each of these bear spectral characteristics not readily reproduced by a single log-normal distribution in spectral space, as is required by the Texel rending. Thus, achieving perceptually similar versions of these textures using the Texel-rendering scheme represents a significant test of the algorithm’s extensibility.

Furthermore, in this work, we investigate the effect of quantizing the spectral components of a Texel-rendering texture on the perceptual outcome. By leveraging the demonstrated inaccuracy in frequency judgement during human vibrotactile perception (20, 21), the discretization of oscillation frequencies available to the rendering algorithm could greatly reduce the amount of data required to store the texture without perceptual effect.

### Optimization techniques

While the *Central Pitch* and *Irregularity*-rendering parameters were previously shown to give texture designers enough flexibility to predictably match various reference fine textures composed of white noise with Gaussian-shaped spectral amplitude (19), navigating the 2D parameter space to recreate any arbitrary virtual texture is a nontrivial task. Modifying the *Central Pitch* and *Irregularity* parameters individually is referred to in this work as *free exploration*, which was found to be a difficult-to-use methodology for users in a texture matching task, especially for those with little experience tuning virtual textures. Accordingly, we devised an assistive human interface based on established optimization algorithms that simplified the required user input while converging on a Texel rendering that the user perceives as a good match for a reference texture.

The use of optimization algorithms in turning virtual texture parameters has previously found success. In Pescara et al. (22), the researchers use an evolutionary optimization algorithm to personalize the vibrotactile signals generated by a wearable device to maximize discrimination and positive sensation in the subject. Notably, the algorithm required only three pieces of preference information from the subject per iteration.

In Pescara et al. (23), the same researchers as Pescara et al. (22) approach the problem of improving vibrotactile signal discrimination by using a genetic optimization algorithm to tune the stimulus to an individual subject’s sensory preferences. While discrimination performance universally improved, the tuning parameters generated by the group of users differed greatly. This approach demonstrates the success of a generative optimization algorithm responsive to preference input from a test subject.

The researchers of Lu et al. (24) combine an evolutionary algorithm with a Generative Adversarial Network (GAN) to produce perceptually matching virtual textures for real reference textures. In this approach, when a user selects the closest match to the reference among a group of virtual textures generated by the GAN, the preference drives the evolutionary algorithm to select optimal texture parameters (input to the GAN) toward matching the reference texture. This process repeats until convergence is detected. This technique effectively approaches the best-match texture parameters using only preference data (in this case, best match to a reference among several candidates), while presenting a substantially simpler task to a user as compared to free exploration of multiple parameter dimensions simultaneously.

## Materials and methods

### Reference textures

**Fig. 4.**
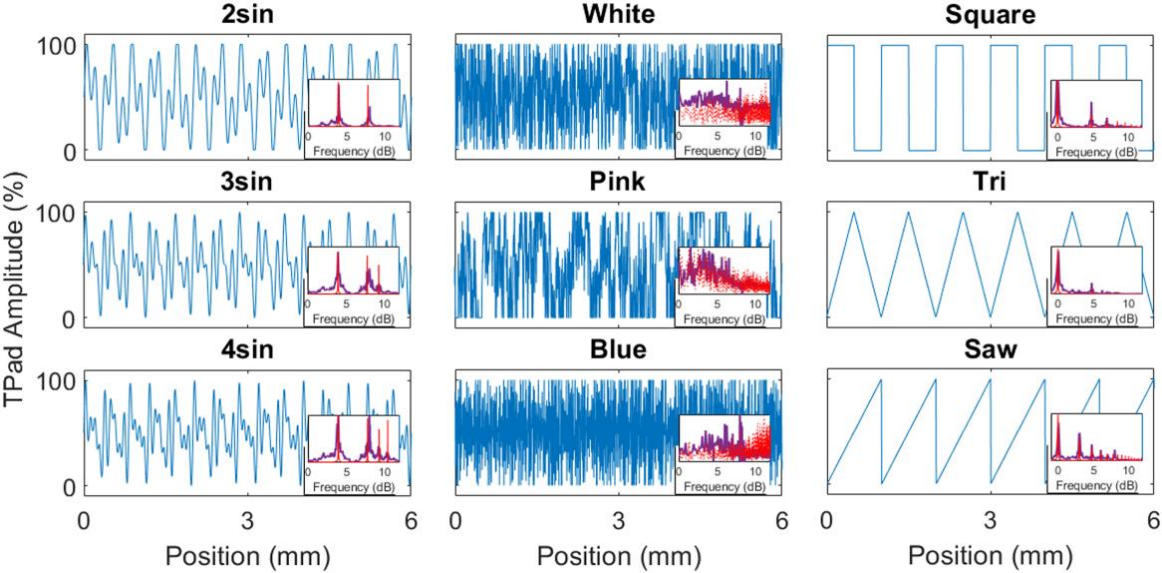
The nine reference textures used in this study represented by 6-mm long samples of the space-domain TPaD texture maps. TPaD amplitude refers to the friction reduction amplitude achieved at any point through ultrasonic vibration between the TPaD surface and the test subject’s finger, scaled by the maximum amplitude of the device. Inset: Fast Fourier Transform magnitude traces for each of the textures, normalized for visibility. Red: commanded friction reduction spectral magnitude; purple: lateral fingertip velocity spectral magnitude acquired via laser Doppler vibrometer. (Note that dB units are calculated with a reference value of 1 cycle/mm).

To test the viability of the Texel-rendering algorithm, a diverse set of multifrequency reference textures was sought, summarized in Fig. 4. Rigorous testing of the algorithm would ideally include textures that do not intuitively lend themselves to successful imitation by a string of single-frequency texels stochastically drawn from a Gaussian distribution (in log-frequency space). To this end, three families of fine textures were identified. Note that all textures were scaled to have the same maximum amplitude.

The first family, *Sums of Sinusoids*, consisted of the equally weighted superposition of two or more sinusoidal components of differing frequencies. This resulted in oscillating, but dramatically nonsinusoidal, textural signals. Care was taken in the choice of the discrete frequencies to avoid beat frequencies below 1 cycle/mm, which would be perceivable as coarse textural features. The first texture in this family was an equally weighted sum of a 2.5 cycles/mm sinusoid with a 6 cycles/mm sinusoid. The second summed 2.5, 6, and 8.5 cycles/mm. The third summed 2.5, 6, 8.5, and 11 cycles/mm. (These frequency values correspond to 4.0, 7.8, 9.3, and 10.4 dB, respectively.) For brevity in figures, these textures are named *2sin*, *3sin*, and *4sin*, respectively.

The second family, *Colors of Noise*, collected three wide-band spectral signals inspired by analogous audio signals of the same name. Specifically, these textures included White Noise (with constant power spectral density across all renderable frequencies), Pink Noise (with power spectral density inversely proportional to frequency), and Blue Noise (with power spectral density proportional to frequency), named in figures *White*, *Pink*, and *Blue*, respectively.

The third family, *Shaped Waveforms*, consisted of three textures where a particular arrangement of spectral amplitude and phase produce oscillating signals with recognizable shapes in the spatial domain. All of these textures were constrained to have a fundamental frequency of 1 cycle/mm. The three waveforms selected were a square wave (containing odd-integer harmonics decreasing at a rate of 6 dB/octave), a triangle wave (containing odd-integer harmonics decreasing at a rate of 12 dB/octave), and a sawtooth wave (containing all integer harmonics decreasing at a rate of 6 dB/octave), named in figures *Square*, *Tri*, and *Saw*, respectively.

In cases where a single representative texture from each family was required to ensure testing was not prohibitively long, the following three were chosen on the basis of pilot studies indicating they were sufficiently dissimilar from one another: The sum of four sinusoids from the Sums of Sinusoids family (*4sin*); Pink Noise from the Colors of Noise family (*Pink*); and the sawtooth wave from the Shaped Waveforms family (*Saw*). These will be called the Family Exemplars.

### Equipment

All testing was performed using a TPaD (25), generating 1D spatial texture maps 101.76 mm long with a resolution of 19,200 discrete points. Texture maps and test subject graphical user interface (GUI) controls were controlled via MATLAB R2021b on an attached PC.

Due to the limitations set by the hardware used, Pitch and Irregularity were limited for all testing to the following ranges. Pitch: 0.01 to 94 cycles/mm (−20 to 19.7 dB); Irregularity: 0.001 to 3.16 cycles/mm (−30 to 5 dB).

To eliminate any contribution toward textural perception made by sound cues, all subjects wore headphones playing Pink Noise audio during all tests and confirmed before testing that the volume level made inaudible any sounds produced by the TPaD equipment.

### Experimental design

#### Free exploration

To study the ability of a user to tune the two-parameter Texel-rendering space to generate a desired tactile effect, the *Free Exploration* test was designed, so-called as it tasked subjects with matching Texel renderings to reference textures by freely modifying the Central Pitch and Irregularity variables of the Texel Rendering (with Texel length held constant at 0.25 mm). Note that, due to the constraint of continuous phase between Texel boundaries, frequencies with period above the Texel length can be rendered. For example, if a continuous series of several Texels are assigned similar frequencies that fall below the Texel length, the oscillation is rendered continuously across these Texels.

**Fig. 5.**
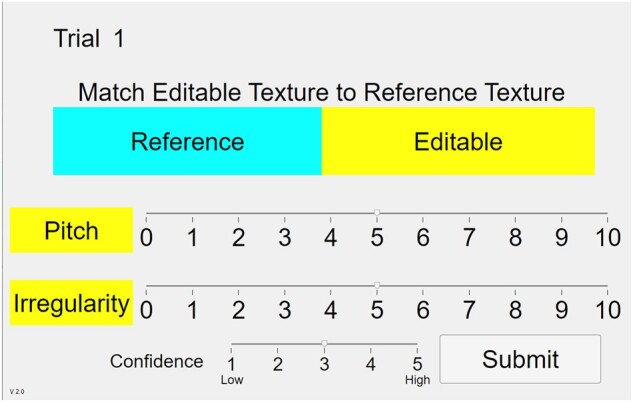
GUI for the free exploration test.

The test sequence was as follows. During each of nine trials, the left half of the TPaD (50.88 mm long) presented one of nine reference textures, while the right half presented the Texel rendering generated by the subject’s current Central Pitch and Irregularity choices. Using two slider controls on the GUI (Fig. 5), the subject was free to alter both Central Pitch and Irregularity with the stated goal of matching the reference texture to the Texel rendering. When the subject was satisfied with the match, confidence in the match was recorded on another slider control from 1 (least confident) to 5 (most confident). Following this, the Central Pitch and Irregularity settings could be submitted and the trial ended. This was repeated for each of the nine reference textures.

In previous work, the stochastic nature of the Texel-rendering algorithm was demonstrated to have little perceptual effect when the Texel length was sufficiently small, effectively transforming a series of stochastically generated vibration frequencies into the sensation of a homogeneous fine texture (19). To further test the robustness of the rendering algorithm to changes in the choices of Texel vibration frequencies, the *Quantization Test* was designed. Here, *quantization* refers to the discretization of available texel pitch choices.

#### Quantization test

Quantization was performed by limiting Texel vibration frequencies to a discrete set of values, effectively reducing the frequency resolution of the texture. In practice, this was achieved by rounding the frequency to the nearest allowed value following its stochastic generation. The amount of quantization was controlled via the distance (in log space) between allowed values. Log-spaced values (rather than linear-spaced values) were chosen to explicitly match the log-normal nature of the underlying distribution itself, which was determined in Burns et al. (19) to perform well for both low-frequency and high-frequency means. For example, if a quantization distance of 1 dB was chosen, frequencies are rounded to the nearest multiple of 1 dB ≈1.26 cycles/mm following stochastic generation. Note that, in the nonquantized state, the frequency resolution is bounded only by the limits of the textural-rendering hardware (in this study, ∼−20 dB ≈0.01 cycles/mm).

**Fig. 6.**
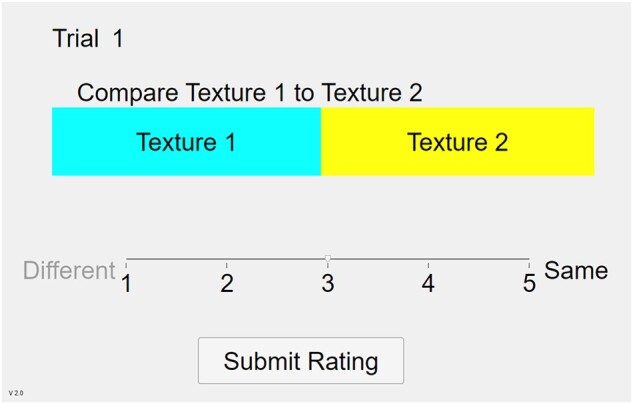
GUI for the quantization test.

The test sequence was as follows. During each of 27 trials, the left and right halves of the TPaD were split to present two different textures. The left half of the display presented one of nine reference textures, while the right half displayed a Texel rendering based either on the test subject’s Central Pitch and Irregularity choices for the matching reference texture (made in the Free Exploration Test) or a Texel rendering based on Central Pitch and Irregularity choices made previously by the *trained rater* (the first author). During the trials based on the three Family Exemplars, the Texel rendering was either nonquantized or quantized to one of three levels: 1, 3, or 5 dB. During each trial, the subject was tasked with rating the similarity between the left (reference) and right (Texel) textures on a scale between 1 (completely different) and 5 (identical), input via a slider control on the GUI (Fig. 6). Following this, the similarity rating could be submitted and the trial ended.

#### Guided exploration test

Based on the results of the Free Exploration test, it was evident that the navigation of the 2D parameter space defined by Central Pitch and Irregularity to accomplish a matching task was difficult for a subject naive to the Texel-rendering algorithm. To alleviate this difficulty, the *Guided Exploration Test* was designed to allow a subject to navigate the parameter space with simple comparison decisions, aided by an assistive algorithm.

**Fig. 7.**
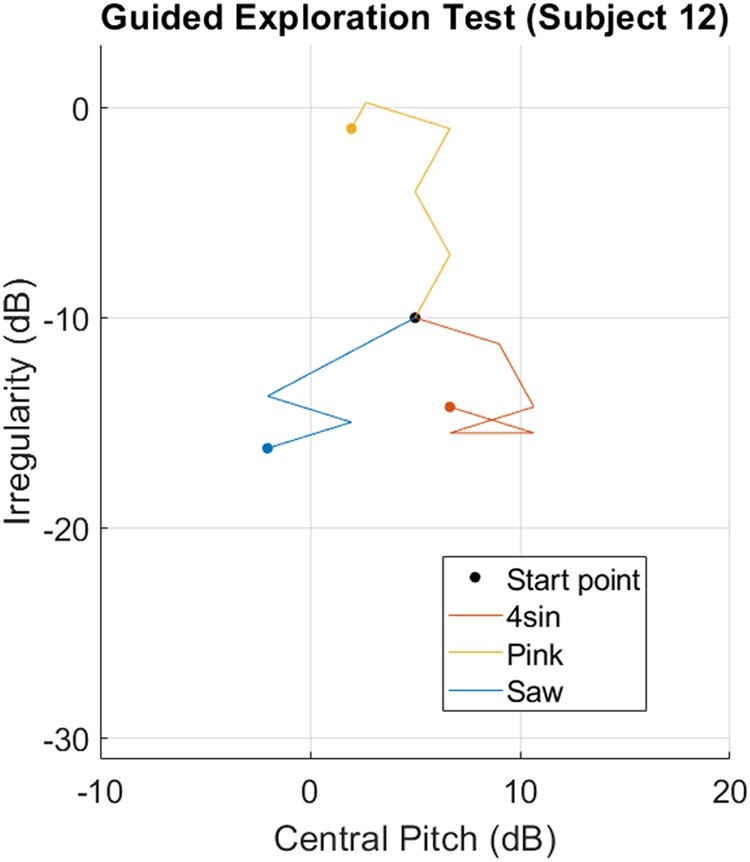
Guided Exploration test results for one subject. Lines connect subspace centroids throughout the optimization algorithm’s progress (line directions match that of the Pursuit Vector for each trial) and solid dots indicate termination points.

The assistive algorithm devised for this study utilized features of the gradient descent procedure (26) and the Nelder–Mead downhill simplex method (27). With the goal of achieving the best perceptual match between some reference texture and a texel rendering, the algorithm searches the 2D texel parameter space (axes: Pitch and Irregularity) using as input comparison data from the human subject, iteratively shrinking the target parameter space until a perceptual match is identified. During any one trial, there is an amount of this 2D space remaining, inside which the ideal perceptual match is believed to exist. The algorithm draws vectors connecting the centroid of this remaining space to its edges along each direction parallel to an axis and along each diagonal. A comparison point is marked at each vector midpoint, totaling eight comparison points surrounding the remaining space’s centroid. Each trial consists of four comparisons, as the subject is asked to choose whether a comparison point or its opposite (the comparison point lying on the parallel vector on the other side of the centroid) feels more similar to a reference texture. The subject can also choose “unsure,” meaning both textures feel equally similar to the reference. The polygon described by the eight comparison points is used analogously to the Nelder–Mead simplex (27), building a set of trial points to establish an ideal direction for optimization over the 2D space. While the simplex generally uses the minimum of three points of function evaluation to establish an approximation of the function’s local behavior, the polygon here uses four “evaluations” (from binary comparisons of eight total points) for this purpose. This redundancy was found to perform well during pilot studies. Based on the trial’s four points of comparison data, the algorithm builds the so-called *Pursuit Vector*: the scaled sum of each vector containing a comparison point deemed “more like the reference texture” (without contribution from each “less like the reference texture” and “unsure” vector). This vector, like the search direction in the gradient descent procedure (26), is an approximation of the direction pointing toward the optimal point (in this case, the texture parameters for an ideal match), and is the direction and magnitude of shift of the remaining parameter space’s centroid at the end of the trial. The Pursuit Vector’s scaling factor was chosen to constrain the vector’s maximum possible magnitude to be half the amount that the remaining parameter space is decreased per trial, which ensured that the remaining parameter space is always a subset of the space in the trial before it. At the end of each trial, the parameter space is decreased and its centroid moves according to the Pursuit Vector. The resulting space is used as the remaining parameter space for the next trial. The optimization algorithm terminates when the remaining space decreases to a single point, and this point is identified as the perceptual match to the reference texture. For algorithmic simplicity and speed of testing, the parameter space decreased by the same amount each trial (in this case, 20% of the initial maximum axes lengths) along both axes, but future versions of this optimization algorithm could utilize adaptive shrink rates as well as convergence criteria not based solely on number of trials. Figure 7 visualizes the progress of the Guided Exploration optimization algorithm throughout the test for one subject.

**Fig. 8.**
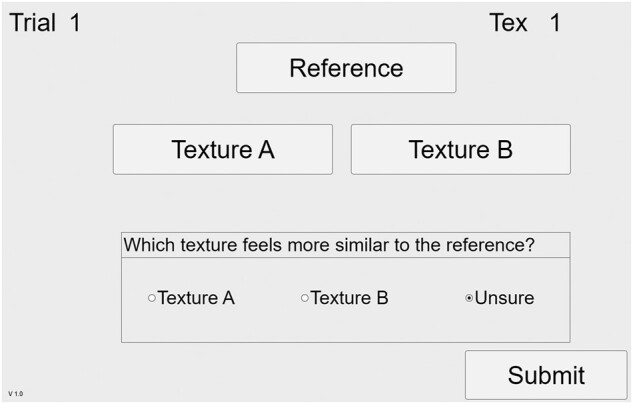
GUI for the Guided Exploration test matching trials.

**Fig. 9.**
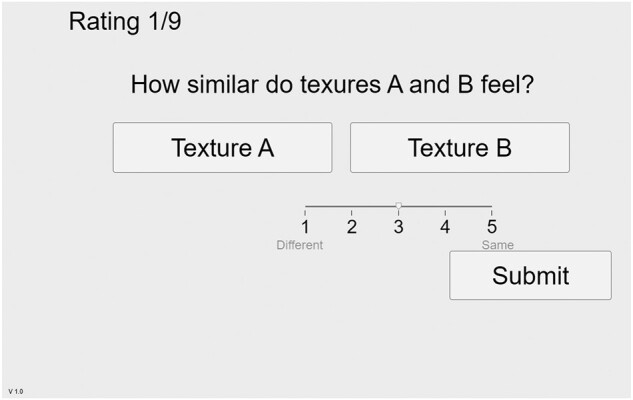
GUI for the Guided Exploration test rating trials.

The test sequence was as follows. For each of three Family Exemplars, 20 comparison trials are conducted. In each trial, the subject is able to freely select the current reference texture and two comparison textures using buttons on the GUI (Fig. 8), causing the TPaD to present the selected texture. After exploring these three textures, the subject confirms which (if either) of the two comparison textures feels more like the reference texture and submits their response, ending the trial. This continues for 20 trials for each of 3 reference textures (totaling 60 comparison trials), after which the user completes 9 rating trials. In each rating trial, the subject is asked to rate the similarity between one reference texture and one Texel rendering. These nine combinations include all permutations of the three reference textures and three Texel renderings based on the parameters identified as ideal matches by the optimization algorithm. The subject enters ratings on a scale between 1 (completely different) and 5 (identical), input via a slider control on the GUI (Fig. 9). Following this, the similarity rating can be submitted and the trial ends.

## Results

There were 18 subjects for the Free Exploration Test, 8 female; 8 subjects for the Quantization Test, 4 female; and 17 subjects for the Guided Exploration Test, 6 female. Six subjects participated in both the Free Exploration Test and Guided Exploration Test. Subjects were instructed to use the index finger of their dominant hand for texture exploration, but any finger on any hand to control the GUI. The protocol was approved by the Northwestern University Institutional Review Board, all subjects gave informed consent, and all subjects were paid for participation.

In all box charts displayed here, filled boxes mark the data between the 0.25 quantile and 0.75 quantile (the interquartile range, or IQR), with the central horizontal line marking the median. Whiskers extend to the nonoutlier minimum and maximum, with outliers (marked as dots) identified as points >1.5 times the IQR away from the top or bottom of the box.

### Texture matching

#### Texel dimensional choices

**Fig. 10.**
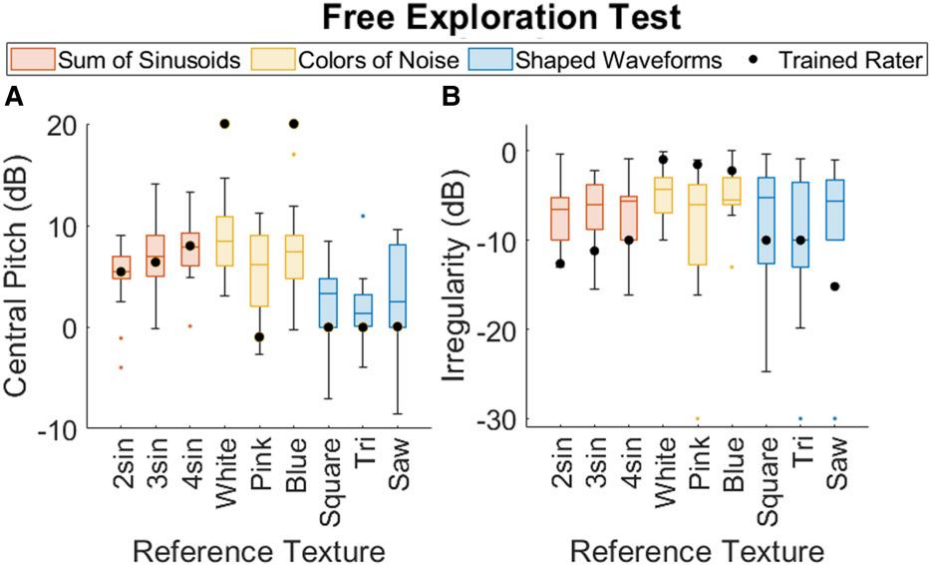
a) Central Pitch and b) Irregularity choices made during the Free Exploration Test, color-coded by texture family. Superimposed points: parameter choices of the trained rater.

**Fig. 11.**
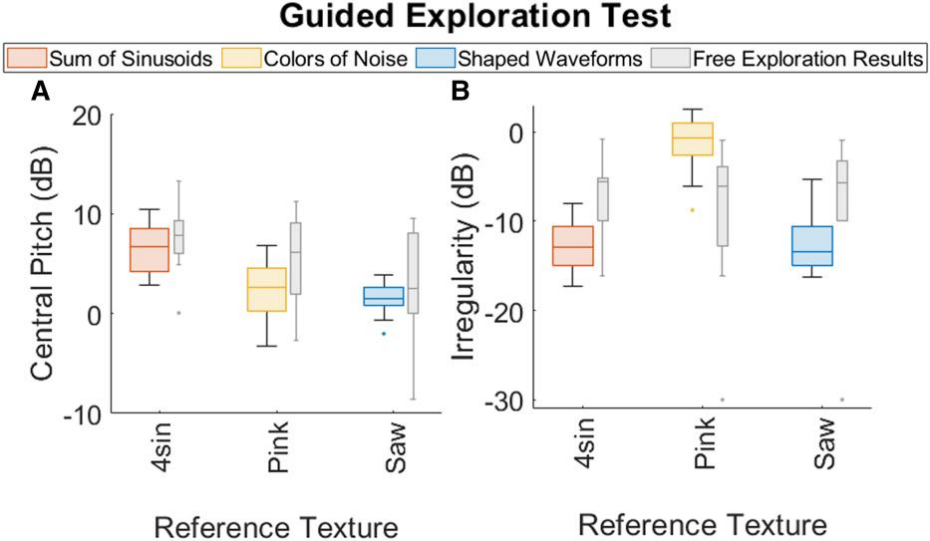
Collected A) Central Pitch and B) Irregularity choices made during the Guided Exploration Test. For comparison, Free Exploration results are superimposed.

Figure 10a and b presents the Central Pitch and Irregularity choices, respectively, made by subjects during the Free Exploration Test, superimposed with the parameter choices of the trained rater. Figure 11a and b presents the same for the Guided Exploration Test. (Note that while the Free Exploration Test used all nine reference textures, the Guided Exploration test used only the three Family Exemplars.)

**Fig. 12.**
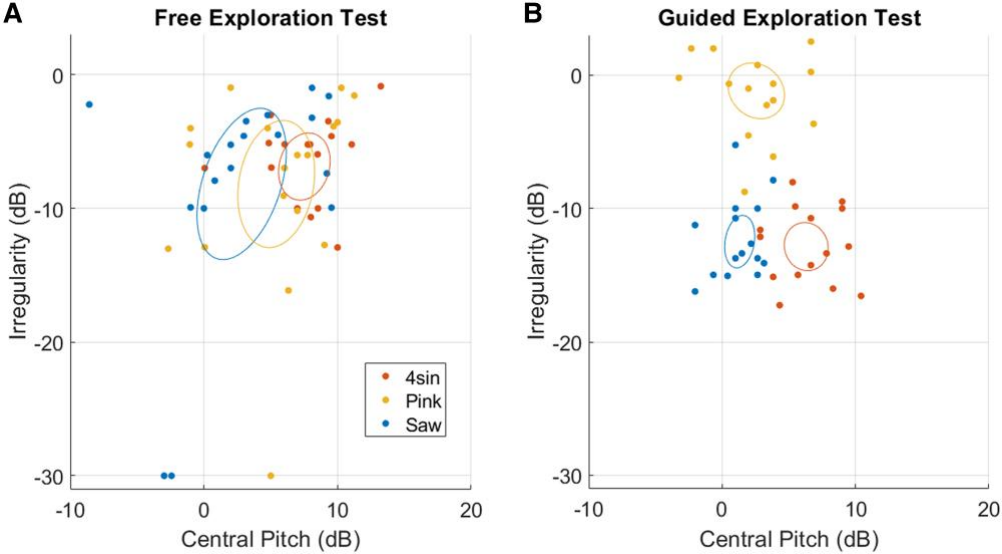
Central Pitch and Irregularity choices made during the A) Free Exploration Test and B) Guided Exploration Test for the Family Exemplars. Superimposed ellipses represent area of 95% confidence around the expected mean of a given texture.

Figure 12a and b presents the parameter choices for the Free Exploration Test and Guided Exploration Test, respectively, on 2D plots, superimposed with 95% confidence ellipses around the expected mean (adapted from Batschelet (28)). Although Free Exploration was tested for all nine reference textures, for ease of comparison with Guided Exploration, we present only the Texel parameters matching the three Family Exemplars for each test.

**Fig. 13.**
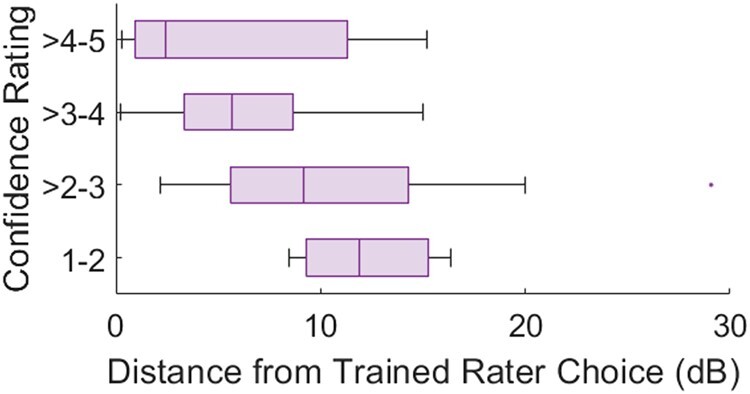
Confidence ratings for all textures made during the Free Exploration Test vs. distance between a test subject’s chosen Texel parameters and those made by the trained rater for the same target reference texture.

Figure 13 summarizes the subjects’ confidence in the Free Exploration Test aggregated across all textures. Confidence rating (divided into four bins) is plotted against distance between the test subject’s Texel parameters and the trained rater’s parameters.

#### Similarity ratings

**Fig. 14.**
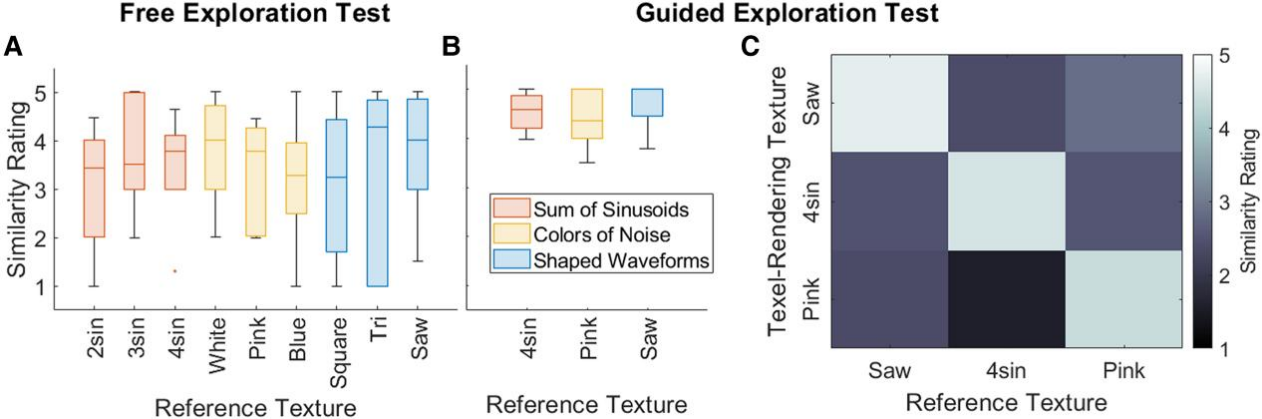
Similarity ratings between reference textures and Texel-rendering matches made during the A) Free Exploration Test and B) Guided Exploration Test. In C), a matrix portrays the similarity ratings made during the Guided Exploration Test between each reference texture (rows) and a Texel-Rendering texture designed to match the named reference texture (columns).

Figure 14a and b presents the similarity ratings made during the Free Exploration Test and Guided Exploration Test, respectively, between the Texel rendering generated by the subject and its matching reference texture. (Note that while the Free Exploration Test used all nine reference textures, the Guided Exploration test used only the three Family Exemplars.) Figure 14c portrays in a matrix the average similarity rating between each reference texture (rows) and the Texel rendering generated by the subject in the Guided Exploration test for the named reference texture (columns).

### Spectral quantization

**Fig. 15.**
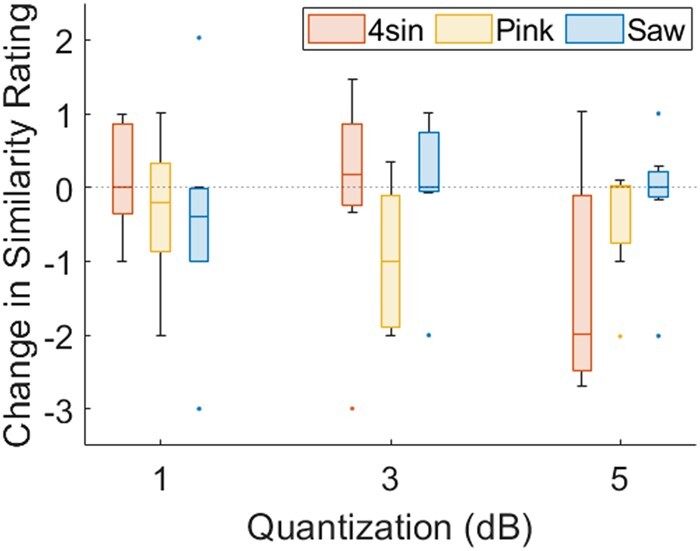
Change in similarity ratings (between reference textures and Texel-rendering matches made during the Free Exploration Test) for each amount of quantization as compared to the similarity rating made in the unquantized state. Superimposed dotted line highlights zero change.

Figure 15 summarizes how pitch quantization affects the efficacy of the Texel rendering, as observed in the Quantization Test. For each of the three Family Exemplars, the effect of quantization is calculated as the similarity rating for a reference texture and its unquantized Texel rendering minus the rating for the same reference texture and its quantized Texel rendering. In the figure, change in similarity rating is plotted for each texture at each of three levels of quantization.

## Discussion

### Parameter tuning through free exploration

The collected Texel parameters chosen during the Free Exploration Test, shown in Fig. 10a and b, suggest that manually tuning Central Pitch (the mean of the underlying distribution) to match a reference represented an easier task than tuning Irregularity (the width of the underlying distribution). This is apparent in the higher variance in Irregularity choices (the height of the box plots in Fig. 10b, average SD: 5.12 dB) as compared to the same in Central Pitch choices (the height of the box plots in Fig. 10a, average SD: 3.78 dB). Such behavior may be due to the fact that Central Pitch is naturally a more familiar perceptual dimension than Irregularity to a naive virtual texture user. While Central Pitch is analogous to the audible pitch of a sound, no such connection exists for Irregularity. Additionally, it is likely that the just-noticeable difference (JND) for Irregularity exceeds that of Central Pitch when both are measured in cycles/mm, but the improved results from the Guided Exploration Test suggest that the variability in Irregularity choices in the Free Exploration Test is affected by factors beyond the threshold of human perception. For the Guided Exploration Test (Fig. 11a and b), average SDs in Central Pitch and Irregularity choices (for all textures) are 2.42 and 2.90 dB, respectively. Additionally, the range of values selected during the Free Exploration test suggests that subjects were capable of tuning Central Pitch more effectively than Irregularity. While the mean value of Central Pitch varied significantly from texture to texture (SD across means: 2.55 dB), the mean value of Irregularity did not (SD across means: 1.54 dB). In short, Irregularity was not effectively used as a texture design parameter during the Free Exploration Test.

The Central Pitch choices made in the Free Exploration Test reveal some notable trends (see Fig. 10a). In the Sums of Sinusoids texture family, the mean value of Central Pitch chosen by subjects follows a rising trend when additional (higher frequency) sinusoids are added, closely matching the Central Pitch choices of the trained rater. This is in line with the results of (20), which demonstrated that the dominant sensation when exploring a texture composed of several discrete frequency components is a single dominant frequency located at the weighted mean of the original components. The Central Pitch choices for this family of textures indeed follow a mean value near such a dominant frequency, increasing as additional frequency components are added to the high end of the spectrum. The sum of 2.5 and 6 cycles/mm (4.0 and 7.8 dB) (*2sin*) produces a mean Central Pitch around 3 cycles/mm (4.8 dB); the sum of 2.5, 6, and 8.5 cycles/mm (4.0, 7.8, and 9.3 dB) (*3sin*) produces a mean Central Pitch around 4 cycles/mm (6.0 dB); and the sum of 2.5, 6, 8.5, and 11 cycles/mm (4.0, 7.8, 9.3, and 10.4 dB) (*4sin*) produces a mean Central Pitch around 6 cycles/mm (7.8 dB).

In the Colors of Noise texture family, the mean Central Pitch choices loosely follow a trend related to the shape of the reference texture’s spectrum: the mean Central Pitch was lower for Pink Noise (which is dominated by low-frequency components) than for Blue Noise (which is dominated by high-frequency components) and White Noise (which has a flat distribution across all frequencies). This suggests that the low-frequency content of a Shaped Noise texture is a more salient perceptual detail than the high-frequency content, dominating the sensation during exploration.

In the Shaped Waveform texture family, mean Central Pitch choices fall close to the fundamental frequency of the waveforms: 1 cycle/mm (0 dB) for all textures. As with the Sums of Sinusoids textures, this is to be expected: the dominant sensation for these textures should indeed be some weighted mean frequency of the components. Unlike the Sums of Sinusoids, however, the Shaped Waveforms are spectrally composed of a single high-amplitude fundamental frequency with many harmonic frequencies with steeply decreasing amplitude (see Fig. 4). The weighted mean, lacking any significant contribution from the harmonic frequencies, remains close to the fundamental frequency, which closely matches the Central Pitch choices made by the trained rater.

Furthermore, the Free Exploration Test results demonstrate that variance in parameter choices, particularly Central Pitch, depend on the reference texture family. The height of the box plots in Fig. 10a demonstrate different average variance in Central Pitch choices per texture family, with average SD values: 3.88 dB for Sums of Sinusoids, 5.63 dB for Colors of Noise, and 3.98 dB for Shaped Waveforms. While the Central Pitch choices for the Sums of Sinusoids and Shaped Waveforms families have lower SD (with means close to the Central Pitch choices made by the trained rater), the Colors of Noise family has a higher SD in Central Pitch choices. This is an expected effect of the perceptual nature of the texture families: while the Sums of Sinusoids and Shaped Waveforms textures are dominated by a few frequency components and lack any wide-bandwidth noise signals, the Colors of Noise textures have no such dominant frequency components. (Compare the spectral signals of the reference textures in Fig. 4.) Effectively, the choice of Central Pitch of the Texel Rendering for a texture in the Colors of Noise family has a smaller perceptual effect than it does for the other texture families. When matching to a high-bandwidth (noisy) texture, it seems to matter more that the Irregularity parameter is high and less that the Central Pitch parameter is located at some precise value. This is not to say that the Central Pitch parameter is unused when matching to noisy textures: note in Fig. 10a that the average Central Pitch values were lower to match Pink Noise than those to match White or Blue Noise.

The Free Exploration Test confidence ratings depicted in Fig. 13 demonstrate that subjects tended to rate their confidence in a match to a reference texture higher when their chosen parameters were close to those chosen by the trained rater. This suggests that the high variance in parameter choices (Fig. 10a and b) and low average similarity ratings between the reference textures and their Free Exploration Test matches (Fig. 14a), mean similarity rating 3.4 out of 5) may not be an inherent weakness of the algorithm but rather a byproduct of the difficulty of the task of navigating the parameter space freely to find a perceptual match to a reference texture. After all, if confidence ratings tend to be higher when Texel renderings use parameters closer to those chosen by the trained rater, it is rational to believe that matches exist but are not easily found by all subjects.

### Parameter tuning through guided exploration

When depicted on a 2D log–log plot as in Fig. 12a and b, the improvement provided by the Guided Exploration Test’s optimization algorithm is apparent. Compared to the parameter choices made in the Free Exploration Test, those made in the Guided Exploration Test exhibit much tighter distributions in both the Central Pitch and Irregularity dimensions, as visualized by the significantly smaller confidence ellipses. It is notable that for the Guided Exploration Test results, the variance in the Irregularity choices (average SD across all textures: 2.90 dB) remains higher than that of the Central Pitch choices (average SD: 2.42 dB), as it did for the Free Exploration Test. This persistent difference, despite the improved method of Guided Exploration, suggests that the limiting factor may be intrinsically perceptual (i.e. a higher JND) for Irregularity than for Central Pitch when both are measured in cycles/mm.

The success of the Guided Exploration Test is further demonstrated in the similarity ratings between reference textures and a Texel rendering utilizing the subject’s chosen matching parameters. While the similarity ratings made during the Free Exploration test did not exhibit particularly high values for all textures (Fig. 14a, mean similarity rating: 3.4 out of 5), those made during the Guided Exploration Test demonstrated high similarity between reference textures and Texel renderings for all textures (Fig. 14b, mean similarity rating: 4.6 out of 5). Additionally, the matrix in Fig. 14c demonstrates that Texel renderings exhibit high similarity ratings only with the reference textures they were tuned to match: all “mismatched” pairings exhibited low similarity ratings (mean similarity rating: 2.1 out of 5). This confirms that the increase in similarity ratings for matches between the Free Exploration Test and Guided Exploration Test were indeed effects of superior perceptual matching and not an effect related to the difference in test methodology (such as the significantly increased test duration in the Guided Exploration Test for each reference texture as compared to that off the Free Exploration Test).

### Pitch quantization

The effects of pitch quantization as shown in Fig. 15 demonstrate several effects. First, it is evident that mean similarity ratings decrease with increasing level of quantization (mean change in similarity rating: −0.23, −0.33, and −0.64 for 1, 3, and 5 dB quantization, respectively). Using a repeated measures ANOVA test, the 4sin texture showed a significantly decreased similarity rating with quantization (P=0.03) and the Pink texture approached significance (P=0.08). The Saw texture, in contrast, was essentially unaffected by the level of quantization (P=0.80).

Further analyses used one-sample *t*-tests comparing the mean change in similarity to zero. A null result is taken to indicate a negligible perceptual impact of quantization at the given level. On this basis it is unlikely that quantization to the 1 dB level has any perceptual effect for any of the textures tested, as the similarity ratings between Texel renderings and reference textures for the unquantized and 1 dB level quantization groups did not approach a statistically reliable difference (P=0.72,0.42, and 0.43 for 4sin, Pink, and Saw textures, respectively). This suggests that 1 dB level quantization is a safe method for texture designers to reduce the required data to store a virtual texture (by greatly reducing the number of possible Texel pitch values) without perceptual effect to the end user. Additionally, it is evident that the effect of quantization is not homogeneous across texture families. Where the Saw texture saw no significant change in similarity rating even at the highest level of quantization, 5 dB, (P=0.72) and the 4sin texture only showed a significant decrease in similarity rating between the unquantized and 5 dB level quantization groups (P=0.05), the Pink texture was much more sensitive to quantization. At the 3 dB quantization level, a significant decrease in similarity ratings is already apparent (P=0.04). Comparing these results to the Central Pitch/Irregularity choices for these textures in Fig. 10a and b, it can be inferred that quantization is less perceptible for textures with lower Irregularity values. This result can be explained by the nature of human vibrotactile perception. An unquantized low-Irregularity texture contains many pitch values close to a mean value. Owing to the lossy nature of human vibrotactile perception, the dominant sensation when exploring this texture would be the some weighted mean frequency of excitation even when multiple frequencies are present, as demonstrated in Friesen et al. (20). When this texture is quantized, it amounts to shifting the closely distributed frequencies to a few, or even a single, value. As the texture is already being dominated by a single-frequency sensation, this shift is perceivable only when the quantized frequency is misaligned with the original dominant frequency by a sufficient margin. On the other hand, an unquantized high-Irregularity texture contains many pitch values across a large frequency band. Both the sensations of a dominant weighted mean frequency and texture *noisiness* are perceived, as demonstrated in Friesen et al. (29). In this case, quantization is more easily perceivable at a lower level: as frequency values are “rounded” to their nearest quantized value, the texture’s dominant frequency may remain stable (as with the low-Irregularity textures), but the *noisiness* sensation is more easily affected. This is likely a result of the wide-band nature of the high-Irregularity textures: as the widely distributed frequency values are quantized, there is more opportunity for concentration in one frequency band that was not originally dominant, producing a perceivable effect. The result suggests that, to guarantee successful quantization, the spectral nature of the texture should first be analyzed: the wider the distribution of frequency values in the original texture, the lower the amount of quantization that can be safely used.

## Conclusion

In this work, the Single-Pitch Texel-rendering algorithm is confirmed to be both flexible: capable of producing close perceptual matches with a wide variety of fine texture families; and practical: with a parameter space navigable by a naive user. Aided by a novel optimization approach, subjects repeatably matched reference textures containing widely varying spectral characteristics with Texel-rendering surrogates, confirming the match through high perceptual similarity ratings. Furthermore, Texel renderings were found to be robust to a level of spectral quantization, exhibiting negligible perceptual effect when Texel pitch values were quantized to steps of 1 dB ≈1.26 cycles/mm. These results suggest that the Texel-rendering algorithm is a strong candidate for the compression of fine texture signals, offering a perceptually rich texture sensation with relatively few input parameters.

Future work will include extending this algorithm to the rendering of coarse textural features as well as standardizing the optimization algorithm of the Guided Exploration Test to better capture convergence behavior during texture exploration.

## Data Availability

The data underlying this article are available in GitHub at https://github.com/NU-Haptics-Lab/SinglePitchTexel.

## Previous Presentation

This text was previously used in the PhD dissertation of the primary author (30).

## References

[1] Culbertson H, Schorr SB, Okamura AM. 2018. Haptics: the present and future of artificial touch sensation. Annu Rev Control Robot Auton Syst. 1(1):385–409.

[2] See AR, Choco JAG, Chandramohan K. 2022. Touch, texture and haptic feedback: a review on how we feel the world around us. Appl Sci. 12(9):4686–2022.

[3] Fettweis GP . 2014. The tactile internet: applications and challenges. IEEE Veh Technol Mag. 9(1):64–70.

[4] Kirci P, Ali-Yahiya T. 2021. 6G for tactile internet. Hoboken, New Jersey: John Wiley & Sons, Ltd. p. 35–64.

[5] Ray PP . 2022. A review on tactile IoT: architecture, requirements, prospects, and future directions. Trans Emerg Telecommun Technol. 33(4):e4428–e4428.

[6] Steinbach E , *et al*. 2019. Haptic codecs for the tactile internet. Proc IEEE. 107(2):447–470.

[7] Hassen R, Guelecyuez B, Steinbach EG. 2020. PVC-SLP: Perceptual Vibrotactile-Signal Compression Based-on Sparse Linear Prediction. IEEE Transactions on Multimedia. 23:4455–4468.

[8] Noll A, Nockenberg L, Gülecyüz B, Steinbach E. 2021. VC-PWQ: Vibrotactile Signal Compression based on Perceptual Wavelet Quantization. 2021 IEEE World Haptics Conference (WHC), Montreal, QC, Canada. p. 427–432.

[9] Okamoto S, Yamada Y. 2013. Lossy data compression of vibrotactile material-like textures. IEEE Trans Haptics. 6(1):69–80. 24808269 10.1109/TOH.2012.18

[10] Hassan W, Abdulali A, Jeon S. 2020. Authoring new haptic textures based on interpolation of real textures in affective space. IEEE Trans Ind Electron (1982). 67(1):667–676.

[11] Basdogan C, Giraud F, Levesque V, Choi S. 2020. A review of surface haptics: enabling tactile effects on touch surfaces. IEEE Trans Haptics. 13(3):450–470. 32340960 10.1109/TOH.2020.2990712

[12] Meyer DJ, Wiertlewski M, Peshkin MA, Colgate JE. 2014. Dynamics of ultrasonic and electrostatic friction modulation for rendering texture on haptic surfaces. In: 2014 IEEE Aptics Symposium (HAPTICS). p. 63–67.

[13] Watanabe T, Fukui S. 1995. A method for controlling tactile sensation of surface roughness using ultrasonic vibration. Proceedings of 1995 IEEE International Conference on Robotics and Automation, Nagoya, Japan. Vol. 1, p. 1134–1139.

[14] Sednaoui T , *et al*. 2017. Friction reduction through ultrasonic vibration part 2: experimental evaluation of intermittent contact and squeeze film levitation. IEEE Trans Haptics. 10(2):208–216. 28222001 10.1109/TOH.2017.2671376

[15] Vezzoli E , *et al*. 2017. Friction reduction through ultrasonic vibration part 1: modelling intermittent contact. IEEE Trans Haptics. 10(2):196–207. 28222002 10.1109/TOH.2017.2671432

[16] Messaoud WB, Vezzoli E, Frédéric G, Lemaire-Semail B. 2015. Pressure dependence of friction modulation in ultrasonic devices. Proceedings of the 2015 World Haptics Conference, Evanston, IL, U.S.

[17] Wiertlewski M, Friesen RF, Colgate JE. 2016. Partial squeeze film levitation modulates fingertip friction. Proc Natl Acad Sci. 113(33):9210–9215. 27482117 10.1073/pnas.1603908113 PMC4995990

[18] Wiertlewski M, Leonardis D, Meyer DJ, Peshkin MA, Colgate JE. 2014. A high-fidelity surface-haptic device for texture rendering on bare finger. In: Auvray M, Duriez C, editors. Haptics: neuroscience, devices, modeling, and applications, Lecture Notes in Computer Science. Berlin: Springer Berlin Heidelberg. p. 214–248.

[19] Burns DA, Klatzky RL, Peshkin MA, Colgate JE. 2022. A low-parameter rendering algorithm for fine textures. IEEE Trans Haptics. 15(1):57–61. 34962881 10.1109/TOH.2021.3138839

[20] Friesen RF, Klatzky RL, Peshkin MA, Colgate JE. 2018. Single pitch perception of multi-frequency textures. 2018 IEEE Haptics Symposium, San Francisco, CA, U.S. p. 290–295.

[21] Martinez JS, Tan HZ, Cholewiak RW. 2021. Psychophysical validation of interleaving narrowband tactile stimuli to achieve broadband effects. 2021 IEEE World Haptics Conference (WHC), Montreal, QC, Canada. p. 709–714.

[22] Pescara E, Hefenbrock M, Rzepka T, Beigl M. 2018. Vibration personalization with evolutionary algorithms. In: Proceedings of the 2018 ACM International Joint Conference and 2018 International Symposium on Pervasive and Ubiquitous Computing and Wearable Computers, UbiComp’18. Singapore: ACM. p. 436–439.

[23] Pescara E, Dreschner F, Marky K, Kunze K, Beigl M. 2020. Genvibe: exploration of interactive generation of personal vibrotactile patterns. In: Proceedings of the Augmented Humans International Conference, AHs’20. New York, NY, U.S.: ACM. p. 1–9.

[24] Lu S, Zheng M, Fontaine MC, Nikolaidis S, Culbertson HM. 2022. Preference-driven texture modeling through interactive generation and search. IEEE Trans Haptics. 15:508–520. 35536794 10.1109/TOH.2022.3173935

[25] Mullenbach J, Shultz C, Piper AM, Peshkin M, Colgate JE. 2013. Surface haptic interactions with a TPaD tablet. In: *Proceedings of the Adjunct Publication of the 26th Annual ACM Symposium on User Interface Software and Technology, UIST’13 Adjunct*. New York (NY), USA: Association for Computing Machinery. p. 7–8.

[26] Cauchy A-L . 1847. Méthode générale pour la résolution des systèmes d’équations simultanées. C R Acad Sci Paris. 25:536–538.

[27] Nelder JA, Mead R. 1965. A simplex method for function minimization. Comput J. 7(4):308–313.

[28] Batschelet E . 1981. Circular statistics in biology. Mathematics in biology. London, New York, Sydney: Academic Press.

[29] Friesen RF, Klatzky RL, Peshkin MA, Colgate E. 2021. Building a navigable fine texture design space. IEEE Trans Haptics. 14:897–906. 34166203 10.1109/TOH.2021.3092077

[30] Burns DA . 2023. A multi-scale, low-parameter rendering algorithm for virtual textures [PhD thesis]. Northwestern University. Evanston, IL, U.S.

